# Photoelectrochemical UV Detector Based on High-Temperature Resistant ITO Nanowire Network Transparent Conductive Electrodes: Both the Response Range and Responsivity Are Improved

**DOI:** 10.3390/nano13142086

**Published:** 2023-07-17

**Authors:** Ying Xiang, Baoping Li, Yitao Fan, Miaomiao Zhang, Wenxuan Wu, Ze Wang, Minghui Liu, Hu Qiao, Youqing Wang

**Affiliations:** 1 College of Mechanical and Electrical Engineering, Shaanxi University of Science and Technology, Xi’an 710021, China; 2 The Youth Innovation Team of Shaanxi Universities, Shaanxi University of Science and Technology, Xi’an 710021, China; 3 School of Mechatronic Engineering, Xi’an Technological University, Xi’an 710021, China

**Keywords:** ITO nanowires, physical template method, photoelectrochemical, high-temperature resistant, response range

## Abstract

UV transparent conductive electrodes based on transferable ITO nanowire networks were prepared to solve the problem of low UV light utilization in conventional photoelectrochemical UV detectors. The mutually cross-linked ITO nanowire network achieved good electrical conductivity and light transmission, and the novel electrode had a transmission rate of more than 80% throughout the near-UV and visible regions. Compared to Ag nanowire electrodes with similar functionality, the chemical stability of the ITO nanowire transparent conductive electrode ensured that the device worked stably in iodine-based electrolytes. More importantly, ITO electrodes composed of oxides could withstand temperatures above 800 °C, which is extremely critical for photoelectrochemical devices. After the deposition of a TiO_2_ active layer using the high-temperature method, the response range of the photoelectrochemical UV detector was extended from a peak-like response between 300–400 nm to a plateau-like response between 200–400 nm. The responsivity was significantly increased to 56.1 mA/W. The relationship between ITO nanowire properties and device performance, as well as the reasons for device performance enhancement, were intensively investigated.

## 1. Introduction

In recent years, photoelectrochemical (PEC) ultraviolet (UV) detectors have attracted extensive research interest because of their advantages, such as a simple process, low energy consumption, and fast response [1,2,3,4,5]. The photoanodes, electrolytes, and counter electrodes that constitute PEC UV detectors have been extensively studied, with the most important research having been conducted on photoanodes, which are composed of transparent conductive electrodes (TCEs) and photoactive layers [6,7,8]. The research on photoanodes has mainly focused on regulating the photoactive layer, on studying materials (such as TiO_2_, SrTiO_3_, CdS/ZnO, etc.), and on nanostructures (nanosheets, nanospheres, nanorod arrays, nano-branched heterojunctions, etc.) [9,10,11]. There is less research on TCEs in PEC UV detectors, but the TCE is sometimes more important as a window into the device than the photoactive layer [12]. The most important properties of the TCE are its light transmittance and electrical conductivity, which entails maintaining good electrical conductivity while achieving high transmittance [13,14]. For UV devices, the TCE needs to have good transmittance over the entire UV–Vis range to ensure that the device has a wide response range. Various types of TCEs that can be applied to PEC UV detectors have been investigated, including metal nanowires (NWs), graphene, nano-patterned metal grids, ZnO/Ag and NWs/ZnO multilayer films, stainless steel nets, semiconductor NWs, and so on [15,16,17,18,19,20,21]. Many schemes have also been studied to improve the optical and electrical properties of the TCE, such as increasing the connectivity through thermal annealing, removing surface organic impurities using laser irradiation, and removing impurities through solution washing [22,23]. At present, most PEC UV detectors use FTO\ITO glass as the TCE, but they have a serious UV filtration problem; that is, they cannot pass through UV light below 300 nm, and there is absorption of the 300–400 nm range of UV light. As a result, conventional PEC UV detectors can only operate in the 300–400 nm range, thus showing a unimodal response curve [3]. To solve this problem, researchers have carried out various research efforts to try to extend the response range of the device. On the one hand, Wei et al. [24] prepared special collector built-in devices based on macroscopic metal braided networks (such as stainless steel mesh and titanium mesh), thereby successfully avoiding the effect of the TCE on UV light. However, the insufficient active material load and specific surface area of the photoanode seriously limited the performance improvement. On the other hand, we prepared a TCE composed of Ag NWs with a high UV transmittance using the physical template method. The use of this electrode can ensure that the UV light reaches the semiconductor active layer efficiently, which plays a positive role in improving the performance of the device. However, the introduction of metal NWs brought a serious problem, which is that the electrode could only withstand temperatures of about 360 °C, thus meaning that the active layer could only be prepared using the low-temperature method with low performance [12]. At the same time, many metallic materials are extremely chemically unstable in iodine-based electrolytes. We believe that, among the many strategies, designing a TCE with a high transmittance in the UV–Vis range that has good stability (a heat resistance temperature greater than 600 °C and that is chemically stable) is the best way to achieve high performance PEC devices. Compared with metal NWs with poor thermal stability, a transparent conductive oxide (TCO, such as FTO and ITO) has good optical properties, mechanical stability, chemical stability, and thermal stability. As mentioned above, a TCO generally does not transmit UV light, and solving this problem is the key to carry out our research. We surmised that the effective idea was to discretize the continuous TCO film.

Here, we prepared a transferable single-layer ITO NW network through a special physical template method, and after covering it on a quartz substrate, we successfully obtained a TCE with a good transmittance in the whole UV band. The ITO NWs act as conductive channels to ensure electron transfer from the electrodes, while the voids in the NWs allow light of any wavelength to pass through efficiently. After optimization, the ITO NW TCE (INTCE) could achieve 80% transmittance in the near UV region (200–400 nm), while the traditional TCEs used for PEC UV detectors cannot transmit UV light below 300 nm. The INTCE also showed good chemical stability and could be used in iodine electrolyte devices. In the case of low transmittance, the fuse temperature of the INTCE reached 800 °C, which is much higher than the annealing temperature of the ordinary active layer. Under the guarantee of excellent heat resistance, a TiO_2_ active layer was prepared using the high-temperature sintering method. The response range of the PEC UV detector using INTCE was widened, and the responsivity was also significantly improved.

## 2. Materials and Methods

**Figure 1:**
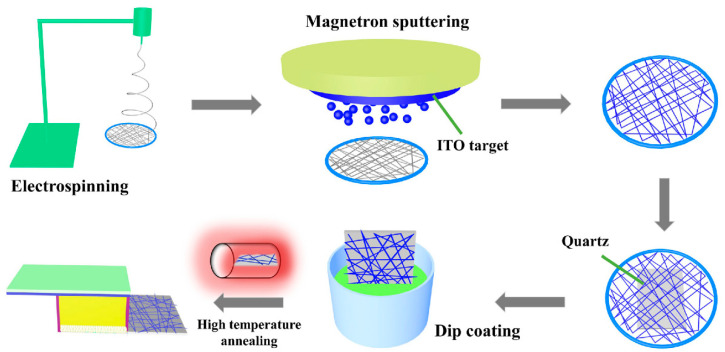
Preparation process of ITO NW networks and PEC UV detector.

All the chemical reagents used in the experiment were analytically pure. The process of preparing the INTCE and PEC UV detectors is shown in Figure 1. The whole experimental flow included (1) electrospinning to prepare PVP NW network templates; (2) magnetron sputtering to deposit ITO layer on the templates; (3) transferring the ITO NW network to the quartz substrate; (4) deposition of the TiO_2_ active layer; (5) device encapsulation; and (6) performance testing.

### 2.1. Preparation of INTCE

We slowly poured 0.7 g of polyvinyl pyrrolidone (PVP) into 10 mL of anhydrous ethanol and magnetically stirred the solution for 15 min. The stirred solution was poured into the needle of an electrospinning device, and we adjusted the distance between the electrospinning needle and the collecting metal ring to 15 cm. The spinning parameters were adjusted to the working voltage of 15 kV, and the spinning time was 30 s; hence, the preparation of the PVP template was completed. At this time, the PVP nanonetwork was covered on the metal ring, thus forming a suspended film. Then, using this as a template, the corresponding nanonetwork could be obtained by depositing the target material on the PVP NWs. The ITO layer was deposited on the PVP template by magnetron sputtering. The parameters of magnetron sputtering were the following: vacuum degree was 8 × 10^−4^ Pa, working pressure was 0.5 Pa, sputtering power was 50 W, deposition time was 10 min, and the sputtering target was the ITO. The quartz glass was cleaned with deionized water and ethanol, and ITO NW network was transferred to the quartz glass. We simply sleeved the metal ring over the substrate when transferring it. Finally, the ITO NWs/quartz was prepared as TCE for PEC UV detector.

### 2.2. Preparation of TiO_2_ Photoactive Layer

(1) Low-Temperature method:

Added 1 mL of acetic acid and 2 mL of deionized water to 30 mL of ethanol. Slowly added 15 mL of titanium isopropoxide to the mixed solution and stirred with magnetic force for 30 min. Placed the mixed solution in a drying oven at 70 °C for 5 h, and then placed the resulting white powder in a muffle furnace at 550 °C for sintering for 2 h. The mixed solution was prepared with 5 mL anhydrous ethanol, 0.5 mL ammonia, and 0.5 mL deionized water. An amount of 1 g TiO_2_ powder was obtained and dissolved in the mixed solution, and the mixed solution was stirred magnetically for 10 min. Then, the prepared solution was dropped onto the INTCE with a pipette, and the INTCE was placed in a drying oven at 80 °C for 30 min.

(2) High-Temperature method:

TiO_2_ photoactive layer was prepared by sol gel method and high-temperature annealing method. Dissolved 2.5 mL of titanium isopropoxide in 10 mL anhydrous ethanol and stirred magnetically for 5 min (solution i). An amount of 2.5 mL acetic acid and 1 mL hydrochloric acid were added to 5 mL ethanol and stirred magnetically for 5 min (solution ii). An amount of 1.5 mL OP emulsifier and 0.3 g PVP were dissolved into 8 mL anhydrous ethanol and magnetically stirred for 15 min (solution iii). The three solutions were mixed together and magnetically stirred for 15 min. The prepared mixed solution was dripped onto the INTCE and transferred to a muffle furnace at an annealing temperature of 550 °C for 2 h.

### 2.3. Encapsulation of the PEC UV Detector

The PEC UV detector consisted of a counter electrode, electrolyte, and photoanode. The preparation process of the counter electrode involved the following: H_2_PtCl_6_ was spin-coated on an FTO substrate and annealed for 20 min in a muffle furnace at 400 °C in atmospheric environment. The electrolyte was located between the photoanode and counter electrode and was composed of 0.1 M lithium iodide dissolved in acetonitrile solution, 0.6 M 1,2-dimethy l-3-propylimidazolium, 0.05 M iodine, and 0.5 M 4-tert-butylpyridine. Finally, the counter electrode, the photoanode, and the electrolyte were packaged into a sandwich-shaped device. The electrolyte filled between the photoanode and the counter electrode and diffused into the gaps of the active layer material to participate in charge exchange.

### 2.4. Characterization

The transmittances of the different TCEs were measured using a UV–Vis spectrophotometer. The heat resistance temperatures of the INTCEs were tested by annealing samples in muffle furnace. The conductivity of the INTCE was characterized by measuring the sheet resistance with four probes. The morphology and structure of the nanomaterials were characterized using scanning electron microscopy (SEM) and X-Ray diffraction (XRD). An electrochemical workstation was used to characterize the photovoltaic performance of devices. The photovoltaic characteristic curve, Nyquist diagram, spectral responsivity curve, and switching performance curve were tested under different wavelength light sources using xenon lamps and monochromators. EIS measurements were carried out in the frequency range from 0.01 Hz to 100 kHz. The time response curves were tested at 0 V bias.

## 3. Results and Discussion

**Figure 2:**
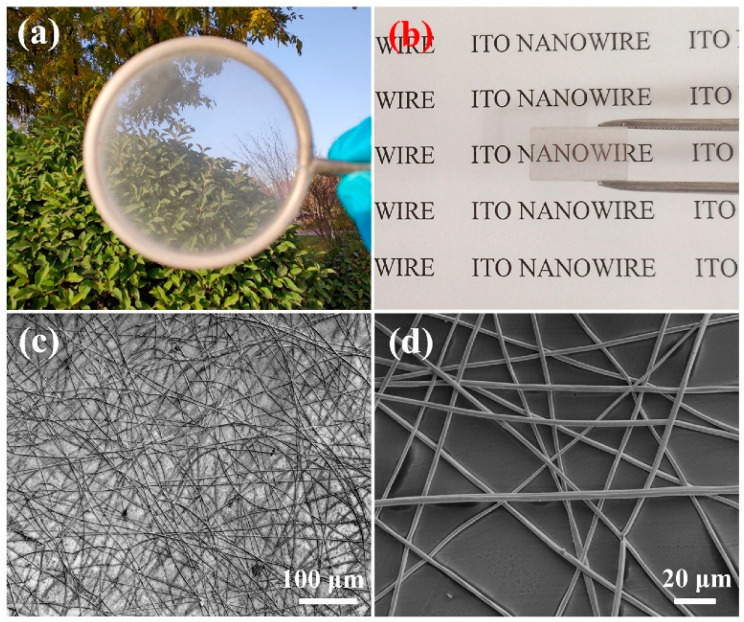
(**a**) Physical image of ITO NW film; (**b**) The INTCE obtained by transferring ITO NW film to a quartz substrate; (**c**,**d**) SEM images of ITO NW networks with different magnification.

The physical image of the ITO NWs prepared on a metal ring using electrospinning and magnetron sputtering methods is shown in Figure 2a. The ITO NWs were evenly distributed on the metal ring, and the entire film was self-supporting and could be easily transferred to any substrate as it was. The nanowire networks are so thin that they are almost invisible. The illustration in Figure 2b is a physical image of the INTCE, which was obtained by transferring the ITO NW networks to a quartz substrate. Figure 2c,d shows the SEM image of the ITO NWs, which overlapped to form a network structure with a single ITO NW having a diameter of about 800 nm. The connected ITO NWs were responsible for transporting electrons. The gaps in the NW networks allowed UV light to pass through without barriers.

**Figure 3:**
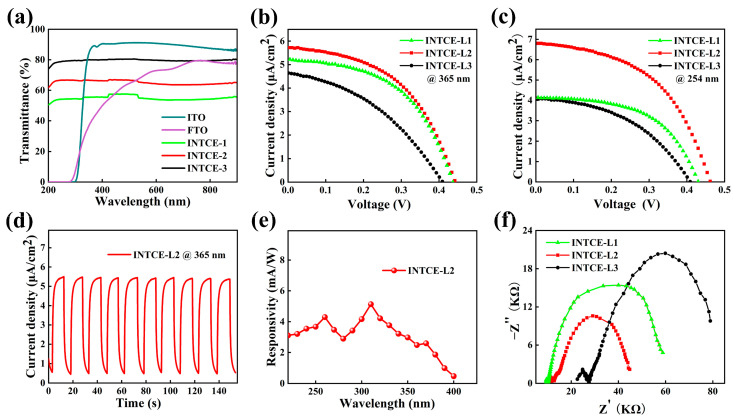
(**a**) Transmission spectra of FTO, ITO, and INTCE at 200 to 850 nm; (**b**,**c**) Photovoltaic performance of devices at 365 and 254 nm (1200 μW/cm^2^), respectively; (**d**) Switching performance of the device; (**e**) Spectral responsivity at 200–400 nm; (**f**) Nyquist diagrams of the devices (254 nm, 1200 μW/cm^2^).

Figure 3a shows that the FTO and ITO had significant UV light filtering problems and could not transmit UV light below 300 nm. Therefore, the UV light below 300 nm cannot reach the photoactive layer, thereby resulting in the failure of conventional PEC UV detectors to respond to this region. Three INTCEs with different densities of ITO NWs were prepared. The transmittance of the three INTCEs at 254 nm were 81.0%, 66.3%, 53.1%, respectively, and the sheet resistances were 11,150 Ω/sq, 4547 Ω/sq, and 2140 Ω/sq, respectively (they are abbreviated as INTCE-L3, INTCE-L2, and INTCE-L1). It can be seen that the INTCEs had stable light transmittance in the wavelength range of 200–850 nm. The maximum transmittance of the INTCE of UV light at 200–400 nm was about 80%. The light transmission and conductivity of the TCEs composed of conductive NWs usually have a synergistic relationship, meaning that the improvement of the light transmission will reduce the conductivity and vice versa. It should be noted that the sheet resistance of the INTCE is close to the internal resistance of the PEC UV detector, which will have an important impact on the device performance, so it is very important to choose the appropriate combination of transmittance and sheet resistance.

The above INTCEs showed excellent UV transmittance, which is very beneficial to broaden the response range of the PEC UV detector. We first deposited a TiO_2_ active layer on the above electrode using the low-temperature method to study its UV response performance. The morphology and structure of TiO_2_ porous films prepared using the low temperature method are shown in Figure S1. As shown in Figure 3b,c, we tested the volt–ampere characteristic curves of the INTCE-Based PEC UV detectors. Whether at 365 or 254 nm of radiation, the devices showed a certain photoelectric conversion performance; it should be noted that the traditional device was not responsive at 254 nm. Among the three devices, the INTCE-L2 featured a moderate transmittance and conductivity, as well as showed the best performance, which was the result of the competition between the light excitation efficiency and carrier collection efficiency. Compared with the INTCE-L2, the INTCE-L1 had better electrical conductivity, but too few photons passed through it, thus making it is difficult to excite enough photogenerated carriers. The INTCE-L3 had a transmittance of up to 80%, but the conductivity was too poor to effectively collect the photogenerated carriers. Electrochemical impedance spectroscopy was used to further analyze the differences in device performance (as shown in Figure 3f). The series impedance of the three devices increased successively, which was mainly due to the linear influence of the impedance of the ITO NWs, which was also consistent with the increasing trend of the impedance of the INTCE. The INTCE-L2 exhibited the lowest interfacial charge transfer impedance and polarization impedance, which was the main reason for its superior performance. The equivalent circuit for the electrochemical impedance testing is shown in Figure S2. As shown in Figure 3d, when the UV light was periodically turned on/off, the device showed good periodic repeatability. Figure 3e shows the response spectrum of the INTCE-L2. As expected, the device showed a stable response in the whole range of 200–400 nm, with a peak response of 5.2 mA/W.

However, the performance results of the above devices were not ideal; at 1200 μW/cm^2^ of irradiation, the response current density was only about 6 μA/cm^2^. The zigzag shape of the switching curve in Figure 3d is another indication of poor performance. In our experience, the switching curve of high-performance devices should be rectangular. This was mainly due to the problems of low-temperature deposition of the semiconductor thin films, which involve the following: (1) a poor crystallinity of the material and a high interfacial resistance, thereby resulting in difficult carrier diffusion; (2) a serious carrier recombination caused by impurities and defects; and (3) poor contact between the semiconductor and electrode. In many PEC fields, the low-temperature method has always been insufficient to obtain high-performance devices. In previous studies, we also obtained a UV TCE with excellent performance using Ag NWs [12], but, because it could only withstand temperatures up to about 360 °C and was extremely unstable in the electrolytic liquid system, it was difficult to apply to PEC UV detectors. Therefore, we further studied the heat resistance of the INTCE in this work and tried to deposit a TiO_2_ active layer using the high-temperature sintering method.

**Figure 4:**
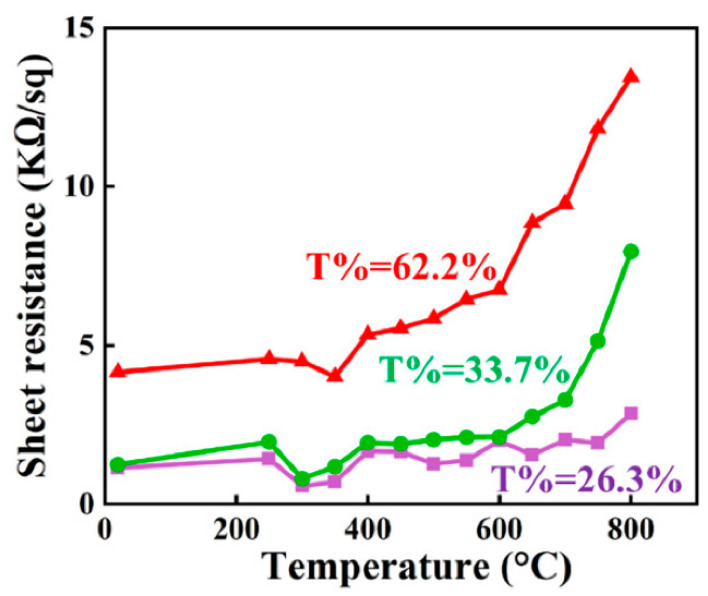
The change in sheet resistance of INTCEs after sintering at different temperatures.

As shown in Figure 4, we selected three INTCEs with different transmittance rates for treatment at different temperatures, and we studied the thermal stability through the change in sheet resistance. When the transmittance of the INTCE was low, that is, when the ITO NWs were relatively dense, the sheet resistance of the film was less affected by thermal treatment, and no significant impedance increase occurred, even at 800 °C. As the ITO NWs became sparser, the heat resistance of the samples with higher transmittance rates gradually decreased. To our satisfaction, the INTCEs could always maintain good thermal stabilities below 600 °C, which was sufficient for the preparation of the TiO_2_ active layers. In addition, compared to metallic materials, ITO NW conductive networks do not have to consider the oxidation problem when exposed to air. It is interesting that the sheet resistance values of the INTCEs showed a paradoxical decrease in the early stage of the heat treatment (around 220 °C). We believe that this was due to the dissolution and decomposition of the PVP template prompting the formation of better links at the intersection of different ITO NWs. This phenomenon also occurred during our previous study of metallic NWs.

**Figure 5:**
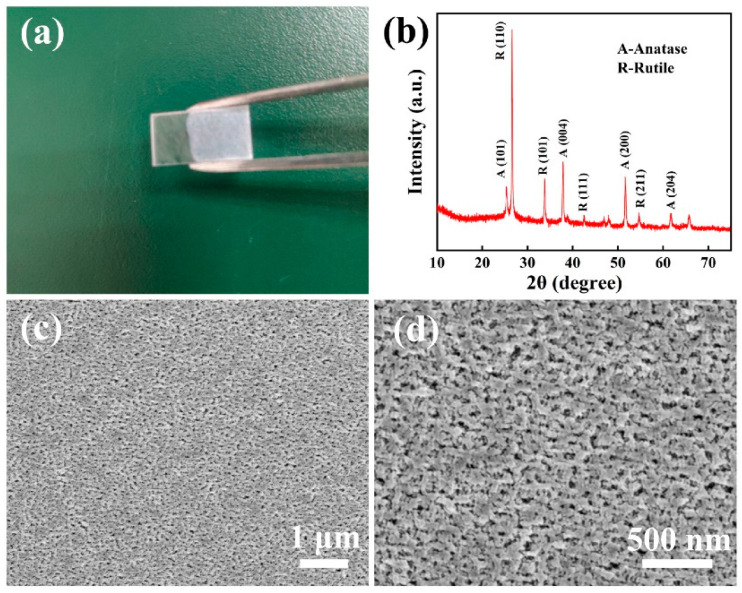
(**a**) Physical image of photoanode; (**b**) XRD image of TiO_2_ photoactive layer prepared using high-temperature method; (**c**,**d**) SEM images of TiO_2_ photoactive layer.

As permitted by good thermal stability, we prepared the TiO_2_ active layers using the sol gel method and through high-temperature sintering at 550 °C. The structure and morphology of the film are shown in Figure 5. As shown in Figure 5a, the left half of sample is the electrode that serves to connect the external circuit and transfer electrons to the external circuit, and the right half is the TiO_2_ photoactive layer that absorbs UV light and produces electron hole pairs. As shown in Figure 5b, the prepared TiO_2_ photoactive layer makes up a mixture of rutile phase and anatase phase. It can be seen from Figure 5c,d that the film prepared by high-temperature sintering was very uniform and dense, and the porous structure facilitated the diffusion of the electrolyte and electron exchange.

INTCEs with different transmittance rates were selected to deposit TiO_2_ active layers using the high-temperature method. The four samples were recorded as INTCE-H1, INTCE-H2, INTCE-H3, and INTCE-H4, respectively, and the corresponding transmittance rates were 18.0%, 42.3%, 61.0%, and 81.8%, respectively.

**Figure 6:**
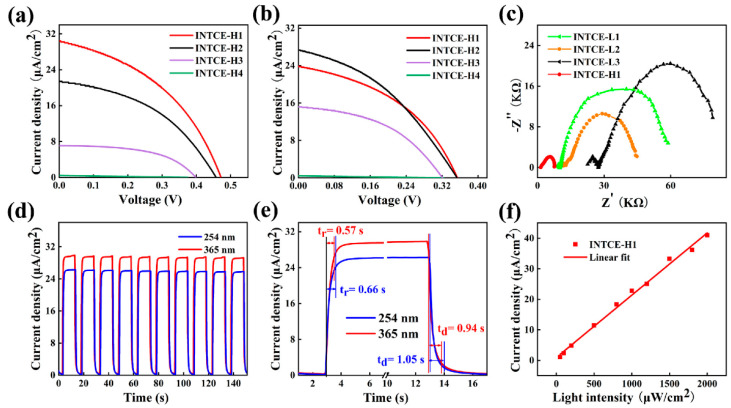
(**a**,**b**) Comparison of photovoltaic performance of devices at 365 and 254 nm, respectively; (**c**) The Nyquist curves of the devices at 365 nm; (**d**) Switching performance results of the devices; (**e**) Response time testing; (**f**) Light intensity response characteristics of the device at 365 nm. The test light intensities were all 1200 μW/cm^2^.

As shown in Figure 6, the performance results of the devices were significantly improved compared with the low-temperature method. When irradiated by UV light with a wavelength of 365 nm, the devices’ performance results decreased as the INTCEs’ transmittance rates increased. When the transmittance rate reached 18.0%, the device performance of the INTCE-H1 was the best, with an open circuit voltage of 0.47 V and a short circuit current density of 30.3 μA/cm^2^. When we tested at 254 nm wavelength radiation, the INTCE-H2 (transmittance rate of 42.3%) showed the best performance, which was slightly higher than the INTCE-H1. Whether tested at 365 or 254 nm, the performance results of the samples gradually decreased with the increase in the transmittance rate, which was consistent with our analysis in the low-temperature test, mainly because the sharp increase in the sheet resistance seriously affected the charge transport. The performance of the device was reduced to almost zero when the transmittance rate reached 81.8% (INTCE-H4), at which point the sheet resistance of the electrode was 22,470 Ω/sq. The significant improvement in device performance illustrates that the preparation of active layers using high-temperature methods is very necessary to obtain high-performance devices. The electrochemical impedance test shows (Figure 6c) that the impedance of the devices prepared using the high-temperature method was substantially reduced, both in terms of series impedance and interfacial charge exchange impedance. As shown in Figure 6d, the switching performance of the device was also significantly improved, with the 0-bias response current showing a distinct rectangular wave shape instead of a sawtooth wave shape under a 10 s switching cycle test. This means that the device had a larger switching ratio with a faster response rate. The response time and recovery time of the detector are labeled in Figure 6e as 0.57 s and 0.94 s (365 nm, 1200 μW/cm^2^), respectively, which were in line with the traditional advantages of PEC UV detectors. Figure 6f shows the good linear light intensity response characteristics of the device.

**Figure 7:**
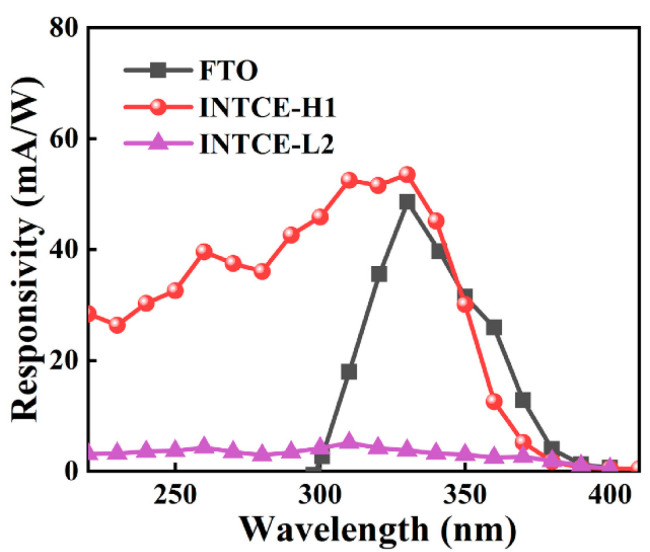
Comparison of the response spectrums of the devices in this work and a conventional FTO device.

Most importantly, we tested the spectral response performance results of PEC UV detectors based on INTCEs and high-temperature sintering methods. For comparison, we prepared a conventional FTO-based device using the same active layer deposition process. As shown in Figure 7, owing to the advantages of the broadband semiconductors, the devices all exhibited good visible light-blind characteristics. The FTO-based device was affected by the light filtering effect and only gave a peak response at 300–400 nm. In line with our expectations, the INTCE-Based PEC UV detectors had a continuous response in the 200–400 nm range and achieved appreciable responsiveness, which is of key importance for the application of the device in the UVB (275–320 nm) and UVC (200–275 nm) ranges. Benefiting from the advantages of the high-temperature method, the response of INTCE-H1 was substantially better than that of the low-temperature device, with a peak response at 332 nm of 56.1 mA/W. This value was also much higher than similar devices based on Ag NW electrodes (15.1 mA/W) [12].

We believe that the synergistic relationship between the light transmission and electrical conductivity of an INTCE can continue to be optimized; for example, this can be achieved by adjusting the diameters of the NWs, thereby improving the connectivity of the NWs and subsequently achieving a more desirable electrical conductivity at a higher transmission rate. The deposition process of the active layer can be referred to from other excellent research results, but this was not the focus of this work. Therefore, an INTCE provides a broad research idea for the continuous improvement of PEC UV detector performance.

## 4. Conclusions

For the problem of the low utilization of UV light sources in conventional PEC UV detectors, we believe that it is an effective idea to use nanoconductive networks through their skeletonization as electrodes. In this work, high UV transmission TCEs based on quartz/ITO NW networks were prepared using a special physical template method. Importantly, this idea effectively solves the problem of the poor thermal and chemical stability of metal NW electrodes, thereby allowing the deposition of a semiconductor active layer through a high-temperature sintering process that ensures that the device can be operated in an iodine-based electrolyte. After optimization, the UV–visible transmittance rate of the INTCE could reach more than 80%, and the heat-resistant temperature exceeded 600 °C and could reach 800 °C under certain conditions. The dense TiO_2_ active layer with good crystallization was prepared using the high-temperature method. Benefitting from the large reduction in the electrochemical impedance and the improvement of the optical absorption performance, the performance of the PEC UV detector was greatly enhanced, with the response range extended from 300–400 nm to 200–400 nm and the magnitude of the responsivity reaching 56.1 mA/W, thus far exceeding that of the low-temperature method applications and previous work.

## Data Availability

The data are contained within the article and supplementary materials.

## Supplementary Materials

The following supporting information can be downloaded at: https://www.mdpi.com/article/10.3390/nano13142086/s1, Figure S1: The photoactive layer prepared using the low-temperature method; Figure S2: Equivalent circuit for electrochemical impedance testing of devices.
