# Supplementary material for: Photoelectrochemical UV Detector Based on High-Temperature Resistant ITO Nanowire Network Transparent Conductive Electrodes: Both the Response Range and Responsivity Are Improved
Source: Nanomaterials (Basel). 2023 Jul 17;13(14):2086. doi: 10.3390/nano13142086 (PMC10383712; doi:10.3390/nano13142086)
Supplement: Supplementary file 1 [file nanomaterials-13-02086-s001.zip › nanomaterials-2492431-supplementary.pdf]

Photoelectrochemical UV detector based on high temperature resistant ITO nano networks transparent conductive electrode: both the response range and responsivity are improved

Ying Xiang [1,#], Baoping Li [2,#], Yitao Fan [1], Miaomiao Zhang [2], Wenxuan Wu [2], Ze Wang [2], Minghui Liu [3], Hu Qiao [3] and Youqing Wang [2,*]

[1] College of Mechanical and Electrical Engineering, Shaanxi University of Science and Technology, Xi’an 710021, China
[2] The Youth Innovation Team of Shaanxi Universities, Shaanxi University of Science and Technology, Xi’an 710021, China
[3] School of Mechatronic Engineering, Xi’an Technological University, Xi’an 710021, China

[#] These authors contributed equally to this work
[*] Corresponding authors. E-mail address: wangyouqing@sust.edu.cn

Supplementary Materials

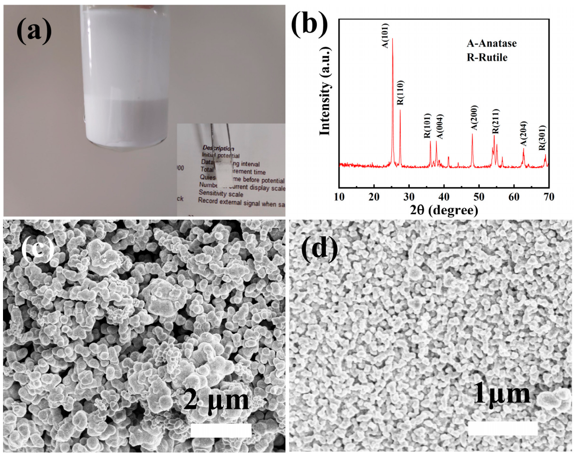

Figure S1. The photoactive layer prepared by low temperature method. (a) Physical image of the solution containing TiO_2 nanoparticles; (b) XRD curve of the TiO_2 photoactive layer; (c, d) SEM images of TiO_2 photoactive layers with different magnification.
